# Hepatic dysfunction and adverse outcomes after total arch repair of acute type a aortic dissection: application of the MELD-XI score

**DOI:** 10.1186/s12872-022-02934-w

**Published:** 2022-11-18

**Authors:** Xinfan Lin, Linfeng Xie, Debin Jiang, Qingsong Wu, Jian He, Liangwan Chen

**Affiliations:** 1 Fujian Medical University, Fuzhou, Fujian 350001 People’s Republic of China; 2 Department of Cardiac Surgery, Union Hospital, Fujian Medical University, Fuzhou, Fujian 350001 People’s Republic of China; 3 Fujian Key Laboratory of Cardio-Thoracic Surgery (Fujian Medical University), Fuzhou, Fujian 350001 People’s Republic of China

**Keywords:** Acute type A aortic dissection, Postoperative hepatic dysfunction, Severe adverse outcomes, MELD-XI score

## Abstract

**Background:**

This study was aimed to investigate the incidence and outcomes of patients with postoperative hepatic dysfunction (PHD) after total arch repair of acute type A aortic dissection, and further explore the risk factors for severe adverse outcomes.

**Methods:**

The clinical data of 227 patients with AAAD treated by modified triple-branched stent graft implantation from January 2020 to January 2021 were collected retrospectively. Including preoperative, surgical and postoperative data. Logistics regression was used to determine the independent risk factors of severe adverse outcomes in postoperative HD patients.

**Results:**

In the early stage after operation, a total of 153 patients were complicated with PHD, accounting for 67.4%. The incidence of severe adverse outcomes in patients with PHD was 43.1%. We found that preoperative moderate/severe pericardial effusion [odds ratio (OR): 11.645, 95% confidence interval (CI): 1.144, 143.617, *P =* 0.045], preoperative imaging data suggest the celiac trunk involvement [OR: 6.136, 95% CI 1.019, 36.930, *P =* 0.048], CPB time > 180 min [OR: 4.855, 95% CI 1.218, 15.761, *P =* 0.034], decreased early postoperative serum albumin [OR: 0.935, 95% CI 0.856, 0.985, *P =* 0.026] were independent risk factors for severe adverse outcomes in patients with PHD.

**Conclusions:**

PHD was associated with increased early mortality and morbidity. Preoperative moderate/severe pericardial effusion, preoperative celiac trunk involvement, cardiopulmonary bypass (CPB) time > 180 min and decreased early postoperative serum albumin were identified as independent risk factors for severe adverse outcomes in patients with PHD.

## Introduction

Acute type A aortic dissection (AAAD) is one of most fatal cardiovascular condition requiring emergency surgery. Without surgery, approximately 50% of patients die within 48 h and 75% of patients within 2 weeks of the onset of symptoms. [1] About one third of all patients with AAAD have preoperative end-organ malperfusion syndromes because of the extensive lesions, which often involve the blood vessels of abdominal organs [2]. Moreover, the operation is technically challenging and associated with surgical trauma, often leading to more postoperative complications[3, 4] Hepatic dysfunction (HD) is a common complication after cardiovascular surgery, as well as one of the important risk factors of poor prognosis. However, there are few studies exploring the impact of postoperative HD (PHD) on clinical outcomes after AAAD surgery. A single-center study has reported PHD significantly increased the incidence of complications and hospital mortality after both coronary artery bypass grafting and valvular surgery. [5] The standard Model for End-Stage Liver Disease (MELD), A logarithmic function of the serum bilirubin, creatinine and international normalized ratio (INR), has been validated as an accurate metric of the degree of liver function. Gorav Ailawadi et al. utilized the MELD score as a method of risk stratification in patients undergoing cardiac surgery, and it proved to be simple and efficient. [6] However, most cardiac patients who requiring surgery, especially aortic disease patients, were managed with systemic anticoagulation before or after surgery, resulting in the elevating INR unable to correctly reflect the hepatic synthetic function. It limits the application of MELD score in the cardiovascular field to a certain extent. To exclude the impact of anticoagulation, a modification of the MELD score (MELD-XI score), has been shown to have the same efficacy as MELD score in predicting short-term survival in patients with cirrhosis. [7] For the complicated steps of repairing aortic arch in the operation of acute aortic dissection, our center used a new technique to repair the total arch by the triple-branched stent graft implantation. [8]Our study was design to apply the MELD-XI score to identify PHD and investigating predictors for severe adverse outcomes occurring after the diagnosis of PHD in patients after aortic dissection repair with triple-branch stent.

## Methods

This was a retrospective study. This study was approved by the institutional review board of our hospital and the need for informed consent was waived.

### Study population

The study group comprised consecutive 227 patients (132 men and 95 women; mean age, 51.1 years; range, 25 to 75 years) who underwent emergent surgery for Stanford type A acute aortic dissection at our institution from January 2020 to January 2021. Patients were on renal replacement therapy before surgery and patients with long-term chronic hepatic dysfunction of any cause before operation were excluded. In addition, patients with postoperative hepatic dysfunction secondary to low cardiac output syndrome (LCOS), hypoxemia and severe infection were also excluded. We recorded the highest MELD-XI score a week after the surgery and divided patients into high-MELD-XI (MELD-XI ≥ 14) and low-MELD-XI (MELD-XI < 14) groups. Severe adverse outcomes that occurred after group assignment were collected, including in-hospital mortality, treated with extracorporeal membrane oxygenation (ECMO), LCOS, malignant arrhythmia, cerebrovascular events, new onset dialysis, re-intubation, tracheostomy, sepsis. All patients were diagnosed as Stanford type A acute aortic dissection by aortic computed tomography angiography (CTA) and treated according to the standard procedure before operation.

## Operative procedure

### Triple-branched stent graft

The triple-branch stent graft independently developed by Professor Chen[8], is a branch-integrated graft composed of self-expanding nickel-titanium alloy stent and polyester vascular graft fabric which Includes one main stent and three sidewall stent grafts.

## Ascending aortic replacement combined with triple-branched stent graft repair

The patient underwent TAR of AAAD through triple-branch stents graft under general anesthesia. Median thoracotomy to expose the surgical field along the ascending aorta and its full length. The operation of aortic root was performed according to the condition of aortic root, valve and coronary ostia, including aortic sinus of Valsalva reconstruction and valve repair or the Bentall procedure. An oblique incision was made near the small bend of the aortic arch. The main part of the triple-branched stent graft implantation was inserted into the true cavity of the aortic arch and the proximal descending aorta, and then three lateral wall grafts were implanted into the corresponding arcuate vessels in turn, then release the bracket. Finally, the end of the triple-branch stent and the artificial polyester blood vessel were anastomosed continuously to complete the operation[8, 9] .

## Diagnostic criteria for postoperative HD

The MELD-XI score was defined as follows: MELD-XI = 5.11* ln (serum bilirubin) + 11.76 * ln (serum creatinine) + 9.44 [10]. Creatinine and total bilirubin were measured as mg/dl, their values less than 1 mg/dl were set to 1 mg/dl to avoid negative number. Creatinine was assigned a value of 4 mg/dl for patients whose creatinine values > 4 mg/dl or in those receiving continuous renal replacement therapy. Recent study showed that the optimal cutoff point of MELD for predicting postoperative mortality is 13.8 in patients undergoing cardiac surgery after liver transplantation [11]. Considering that AAAD surgery is most technically challenging operation in Cardiovascular surgery, we recorded the highest MELD-XI score a week after the surgery and divided patients into high-MELD-XI (MELD-XI ≥ 14) and low-MELD-XI (MELD-XI < 14) groups.

## Statistical analysis

SPSS version 22.0 was used for all statistical analyses. Data are presented as mean ± standard deviation, median [interquartile range], or number (%). The unpaired student’s t test or Mann–Whitney test was performed to compare the continuous variables between groups, and the Chi-square test or Fisher test was used for categorical variables. Significant factors on univariate analysis and clinical factors reported (*P* value less than 0.20) as significant risk factors for severe adverse outcomes were included in multivariate regression analysis, and further multivariate logistics regression analysis was performed to determine the independent risk factors. *P <* 0.05 was considered as statistically significance for all comparisons. The best cut-off value of MELD-XI for predicting severe adverse outcomes in patients with hepatic dysfunction was detected by receiver-operating characteristic (ROC) curve analysis.

## Results

Finally, a total of 227 eligible AAAD patients were enrolled in this study with a median age of 51.1 years old (range 25–75), and 132 (58.1%) of these patients were men. The hospital mortality rate in patients undergoing TAR with triple-branched stent graft was 9.69%, which was almost comparable with the rate reported in previous studies [12]. A total of 153 cases (67.4%) met the PHD's diagnostic criteria (MELD-XI ≥ 14) for the early-stage biochemical markers after operation, which the in-hospital mortality rate was 12.4%. The incidence of severe adverse outcomes in patients with PHD was 43.1%.

### Patients with or without postoperative HD (diagnosed by MELD-XI score)

The proportion of patients with renal insufficiency and imaging data suggest celiac trunk involvement before operation in PHD group was higher, and there was significant difference between two groups. As for preoperative biochemical data, there were significant differences the two groups in terms of albumin, total bilirubin, alanine transaminase and aspartate transaminase upon admission (Table 1). For the operative data, total operation time, CPB time and aortic cross-clamp time (ACC) were significantly different between the two groups (Table 2). The mechanical ventilation time, the ICU stay time and postoperative hospital stays were significantly prolonged in PHD patients. Furthermore, we observed that the proportion of severe adverse outcomes occurring in PHD was much higher than patients without PHD in the same period, including more tracheostomy rate, ECMO support, CRRT treatment, LCOS and worse in-hospital mortality (Table 3).

**Table 1:** Preoperative data of the two groups

Valuables	MELD-XI < 14 (*n =* 74)	MELD-XI ≥ 14 (*n =* 153)	*P* value
**Demographic and baseline risks**			
Age (years)	51.22 ± 11.79	51.37 ± 10.56	0.923
BMI (kg/m^2^)	25.04 ± 3.26	25.29 ± 3.16	0.581
Male gender (n, %)	45 (60.8)	87 (56.9)	0.572
Hypertension (n, %)	54 (73.0)	120 (78.4)	0.362
Diabetes mellitus (n, %)	5 (6.8)	7 (4.6)	0.710
Marfan syndrome (n, %)	5 (6.8)	5 (3.3)	0.392
Smoking history (n, %)	36 (48.6)	83 (54.2)	0.428
Drinking history (n, %)	12 (16.2)	27 (17.6)	0.789
Preoperative LVEF (%)	64.73 ± 5.92	63.17 ± 7.44	0.117
**Preoperative comorbidities**			
COPD (n, %)	1 (1.4)	3 (2.0)	1.000
Shock (sb*P <* 80 mmHg) ^a^ (n, %)	1 (1.4)	6 (3.9)	0.522
Moderate/severe pericardial effusion ^b^ (n, %)	1 (1.4)	9 (5.9)	0.225
Myocardial ischaemia^c^ (n, %)	1 (1.4)	7 (4.5)	0.395
Multi-organ malperfusion ^d^ (n, %)	0 (0)	1 (0.7)	1.000
Renal insufficiency ^e^ (n, %)	7 (9.5)	35 (22.9)	0.023
Acute aortic regurgitation (n, %)	20 (27.0)	44 (28.8)	0.786
Involving the celiac trunk ^f^ (n, %)	4 (5.4)	33 (21.6)	0.002
Involving the renal artery (n, %)	9 (12.2)	25 (16.3)	0.408
**Preoperative biochemical data**			
Leucocytes(10^9^/L)	11.38 ± 3.19	12.36 ± 4.00	0.065
Hemoglobin(g/L)	130.53 ± 18.13	130.88 ± 19.71	0.898
Albumin (g/L)	39.10 ± 8.45	37.13 ± 5.60	0.038
Total bilirubin(U/L)	15.68 ± 8.50	20.96 ± 20.24	0.033
Alanine transaminase (U/L)	31.34 (14.75,38.00)	119.41 (19.00,57.50)	0.003
Aspartate transaminase (U/L)	45.74 (18.50,33.00)	177.41 (21.00,80.00)	< 0.001
γ-glutamyl transferase (U/L)	39.22 ± 42.93	47.08 ± 49.81	0.246

Data are presented as mean ± standard deviation, median [interquartile range] or number (%). Chi-square test for categorical variables and t test or Wilcoxon rank sum test for continuous variables

*Sbp* Systolic blood pressure, *LVEF* Left ventricle ejection fraction, *BMI* Body mass index, *COPD* Chronic obstructive pulmonary disease

^a^Shock:Simply the systolic blood pressure less than 90 mmHg

^b^Moderate/severe pericardial effusion: intraoperatively confirmed effusion volume exceeding 300 ml and were based on cardiac color ultrasonography, an anechoic area in the left ventricular posterior wall were increased(> 10 mm)

^c^Myocardial ischaemia: myocardial ischaemia indicated by the preoperative electrocardiogram, increased myocardial enzymes and segmental wall motion abnormalities revealed by the UCG

^d^Multi-organ malperfusion: clinical symptom or evidence of ischaemia involving three or more organs of the heart, brain, spinal, liver, spinal cord, or gastrointestinal tract and extremities

^e^Preoperative renal insufficiency: Serum creatinine > 130 mmol/L

^f^Involving the celiac trunk: Preoperative abdominal aortic CTA revealed dissection of the celiac trunk

**Table 2:** Surgical data of the two groups

Valuables	MELD-XI < 14 (*n =* 74)	MELD-XI ≥ 14 (*n =* 153)	*P* value
**Intraoperative time**			
Total operation time (min)	285.0 (247.3, 302.5)	315.4 (264.5, 340.0)	0.003
CPB time (min)	133.9 (115.8, 148.5)	155.6 (122.0, 167.0)	0.002
ACC time(min)	42.7 (30.0, 51.0)	53.5 (36.0, 60.0)	0.002
SBP time (min)	9.0 ± 2.6	9.4 ± 3.6	0.337
DHCA time (min)	2.7 ± 1.5	3.0 ± 2.1	0.230
**Intraoperative blood transfusion**			
Erythrocytes (U)	4.24 ± 2.14	4.55 ± 2.28	0.330
Plasma (ml)	483.11 ± 311.35	554.84 ± 293.59	0.092
Apheresis platelets(U)	0.59 ± 0.44	0.60 ± 0.45	0.930

Data are presented as mean ± standard deviation, median [interquartile range] or number (%). Chi-square test for categorical variables and Wilcoxon rank sum test for continuous variables

*CPB* Cardiopulmonary bypass, *ACC* Aortic cross-clamp, *SBP* Selective cerebral perfusion, *DHCA* Deep hypothermic circulatory arrest

**Table 3:** Postoperative characteristics of the two groups

Valuables	MELD-XI < 14 (*n =* 74)	MELD-XI ≥ 14 (*n =* 153)	*P* value
**Postoperative outcomes**			
Mechanical ventilation time (h)	39.4 ± 67.5	65.3 ± 83.2	0.020
ICU stay tine (day)	5.4 (2.0,5.0)	8.5 (3.0,9.0)	< 0.001
24-h postoperative drainage volume (mL)	453.9 ± 316.6	518.8 ± 355.3	0.183
Postoperative hospital stays(d)	17.9 (13.0,20.0)	21.0 (13.0,25.0)	0.046
**Severe adverse outcomes**			
Sepsis (n, %)	6 (8.1)	22 (14.4)	0.178
Nervous system complications ^h^ (n, %)	11 (14.9)	34 (22.2)	0.192
Low Cardiac Output Syndrome ^i^ (n, %)	5 (6.8)	29 (19.0)	0.016
Ventricular fibrillation (n, %)	1 (1.4)	4 (2.6)	0.154
Reintubation (n, %)	4 (5.4)	4 (2.6)	0.493
Tracheostomy (n, %)	4 (5.4)	22 (14.4)	0.047
ECMO (n, %)	1 (1.4)	15 (9.8)	0.020
CRRT (n, %)	5 (6.8)	28 (17.6)	0.021
In-hospital mortality (n, %)	3 (4.0)	19 (12.4)	0.046

Data are presented as mean ± standard deviation, median [interquartile range] or number (%). Chi-square test for categorical variables and t test or Wilcoxon rank sum test for continuous variables

*ICU* Intensive care unit, *CRRT* Continuous renal replacement therapy, *ECMO* extracorporeal membrane oxygenation

^h^Nervous system complications: unconsciousness, delirium, coma and other temporary neurological dysfunctions; cerebral infarction; paraplegia or paraparesis

^i^Low Cardiac Output Syndrome: the need for two or more inotropic medications to maintain systolic blood pressure greater than 90 mmHg or cardiac output > 2.2L/min/m^2^ after adjusting preload and correcting all electrolyte or blood gas abnormalities

### Outcomes of postoperative HD patients

On univariate analysis of the risk factors for severe adverse outcomes occurring in PHD patients, significant preoperative risk factors were hypertension, moderate/severe pericardial effusion, imaging data suggest the celiac trunk and the renal artery involvement. Significant intraoperative risk factors were total operation time, CPB time and ACC time. As well as significant early postoperative risk factors were platelet, albumin, total bilirubin, alanine transaminase, aspartate transaminase, alkaline phosphatase, serum creatinine, lactic acid and 24-h postoperative drainage volume (Table 4).

**Table 4:** Univariate analysis of the risk factors for severe adverse outcomes occurring after the diagnosis of PHD

Valuables	Group A (*n =* 87)	Group B (*n =* 66)	*P* value
**Preoperative factors**			
Age (years)	51.26 ± 10.44	51.50 ± 10.78	0.892
BMI (kg/m^2^)	25.14 ± 3.23	25.50 ± 3.08	0.484
Hypertension (n, %)	61 (70.1)	59 (89.4)	0.004
Diabetes mellitus (n, %)	2 (2.3)	5 (7.6)	0.247
Moderate/severe pericardial effusion (n, %)	1 (1.1)	8 (12.1)	0.012
Acute aortic regurgitation (n, %)	24 (27.6)	20 (30.3)	0.713
Preoperative renal insufficiency (n, %)	11 (12.6)	24 (36.9)	< 0.001
Myocardial ischaemia (n, %)	2 (2.3)	5 (7.6)	0.247
Involving the celiac trunk (n, %)	12 (13.8)	21 (31.8)	0.007
Involving the renal artery (n, %)	11 (12.6)	14 (21.2)	0.156
Preoperative LVEF (%)	63.40 ± 7.19	62.87 ± 7.81	0.661
**Intraoperative factors**			
Total operation time (min)	301.33 (260.00,329.00)	334.00 (272.25,366.75)	0.008
CPB time (min)	144.05 (117.00,162.00)	166.24 (130.00,173.75)	0.009
CPB time > 180 min	9 (10.3)	16 (24.2)	0.021
ACC time(min)	48.97 (35.00,56.00)	57.14 (37.75,68.75)	0.170
SBP time (min)	9.29 ± 3.72	9.57 ± 3.39	0.638
DHCA time (min)	3.07 ± 1.72	2.79 ± 1.45	0.286
Erythrocytes (U)	4.37 ± 2.26	4.77 ± 2.31	0.286
Plasma (ml)	4.37 (2.00,6.00)	4.77 (3.00,6.00)	0.200
Apheresis platelets(U)	0.60 ± 0.45	0.59 ± 0.47	0.815
**Early postoperative factors**			
Leucocytes (10^9^/L)	13.94 ± 3.77	13.95 ± 4.02	0.983
Hemoglobin (g/L)	126.74 ± 17.44	123.08 ± 22.28	0.256
Platelet (10^9^/L)	116.10 ± 55.08	96.70 ± 47.17	0.023
Total bilirubin (U/L)	54.08 ± 54.00	43.41 ± 29.37	0.149
Albumin (g/L)	39.48 ± 6.49	36.98 ± 6.81	0.022
Alanine transaminase (U/L)	135.74 (21.00,55.00)	335.33 (22.75,203.25)	0.187
Aspartate transaminase (U/L)	414.94 (52.00,130.00)	890 (57.75,647.50)	0.021
γ-glutamyl transferase (U/L)	36.36 (14.00,39.00)	30.35 (17.00,36.25)	0.730
Alkaline phosphatase (U/L)	55.98 ± 25.05	48.29 ± 16.27	0.032
Serum creatinine (μmoI/L)	154.33 (104.00,188.00)	227.65 (132.75,276.25)	< 0.001
Lactic acid (mmol/L)	3.93 (1.60,5.40)	5.31 (2.27,7.13)	0.027
24-h postoperative drainage volume (mL)	403.42 ± 264.35	605.03 ± 398.80	< 0.001

Biochemical data are the maximum values within one week after surgery

Group A: No severe adverse outcomes occurrence after diagnosis of postoperative HD. Group B: severe adverse outcomes occurrence after diagnosis of postoperative HD

On multivariate analysis, independent risk factors for severe adverse outcomes were preoperative moderate/severe pericardial effusion [odds ratio (OR): 11.645, 95% confidence interval (CI): 1.144, 143.617, *P =* 0.045], preoperative imaging data suggest the celiac trunk involvement [OR: 6.136, 95% CI 1.019, 36.930, *P =* 0.048], CPB time > 180 min [OR: 4.855, 95% CI 1.218, 15.761, *P =* 0.034], decreased early postoperative serum albumin [OR: 0.935, 95% CI 0.856, 0.985, *P =* 0.026] (Table 5).

**Table 5:** Multivariate analysis of the risk factors for severe adverse outcomes occurring after the diagnosis of PHD

Valuables	Multivariable analysis	
	**P**	**OR (95%CI)**
**Preoperative factors**		
Hypertension (n, %)	0.083	2.741 (0.875,8.581)
Moderate/severe pericardial effusion (n, %)	0.045	11.645 (1.144, 143.617)
Preoperative renal insufficiency (n, %)	0.657	1.390(0.324, 5.954)
Involving the celiac trunk (n, %)	0.048	6.136(1.019, 36.930)
Involving the renal artery (n, %)	0.062	0.128 (0.015, 1.113)
**Intraoperative factors**		
Total operation time (min)	0.087	1.008 (0.999, 1.018)
CPB time (min)	0.933	0.999 (0.981, 1.018)
CPB time > 180 min	0.034	4.855 (1.218, 15.761)
ACC time(min)	0.702	0.996 (0.974, 1.018)
**Early postoperative factors**		
Platelet (10^9^/L)	0.226	1.007 (0.995, 1.019)
Total bilirubin (U/L)	0.994	1.000 (0.991, 1.009)
Albumin (g/L)	0.026	0.935 (0.856, 0.985)
Alanine transaminase (U/L)	0.202	1.002 (0.999, 1.005)
Aspartate transaminase (U/L)	0.182	0.999 (0.999, 1.000)
Alkaline phosphatase (U/L)	0.148	0.980 (0.954, 1.007)
Serum creatinine (μmoI/L)	0.051	1.007 (1.001, 1.014)
Lactic acid (mmol/L)	0.841	0.983 (0.834, 1.159)
24-h postoperative drainage volume (mL)	0.058	1.002 (1.001, 1.003)

Those factors *P <* 0.200 in univariate model were involved in multivariate model

*OR* Odds ratio, *CI* Confidence interval

**Fig. 1:**
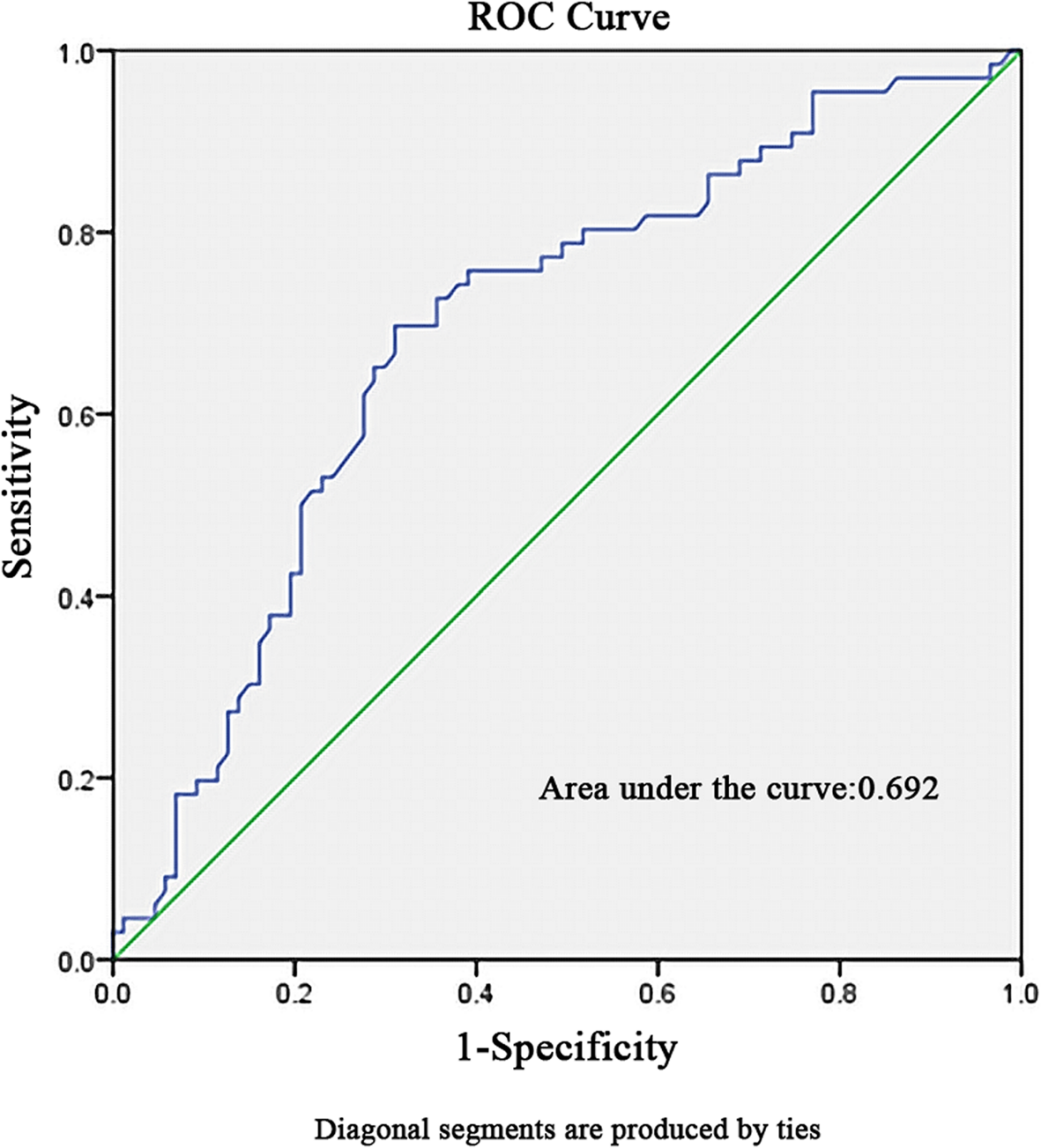
Receiver operating characteristics curve for determination of the cut-off value of MELD-XI score for predicting severe adverse outcomes in patients with PHD

The ROC curve was plotted and the best cut-off value of MELD-XI score for predicting severe adverse outcomes in patients with PHD were 20.82. The value was associated with a sensitivity of 69.7% and a specificity of 31.0%, the area under curve (AUC) was 0.692 (95% CI 0.607–0.777, *P <* 0.001). (Fig. 1).

## Discussion

Adverse clinical outcome in patients with AAAD was affected by several risk factors, and PHD is one of the major factors for poor postoperative prognosis. The cause of.

PHD may be primary liver function damage, or it may be late postoperative complications secondary to LCOS, hypoxemia and severe infection. All the cases of PHD in this study appeared as independent complication diagnosed by the objective and practical MELD-XI score. Then early clinical outcomes of PHD patients were observed. To identify high-risk groups, we focused on preoperative, intraoperative and early postoperative variable of AAAD patients. The results showed that preoperative moderate/severe pericardial effusion, preoperative celiac trunk involvement, CPB time > 180 min and decreased early postoperative serum albumin were independent risk factors for severe adverse outcomes in patients with PHD.

The results of this study show that patients with PHD after AAAD surgery are more likely to have a poor prognosis if the pericardial effusion was found before surgery. Preoperative pericardial effusion is mostly caused by aortic intimal tear, inflammatory exudation or aortic root blood oozing into the pericardial cavity which puts pressure on the heart. [13] More than moderate pericardial effusion before operation has a significant effect on the circulatory system and systemic hemodynamics, which can easily cause pericardial tamponade and poor perfusion of multiple organs, including the heart and liver. [14] At the same time, Due to the impact of surgery, patients with preoperative pericardial effusion are prone to experience significant cardiac insufficiency after operation, which further aggravates the insufficient hepatic perfusion. The whole-body tissue is in a state of acidosis due to insufficient perfusion, and lactate production was markedly increased. However, insufficient perfusion reduces the metabolic capacity of the liver, the postoperative clearance rate of lactic acid decreases. [15] The production of lactic acid accumulation and the metabolism decreases, resulting in hyperlactic acidemia, which reduce myocardial contractility and peripheral vascular sensitivity to catecholamines. Ultimately, the patient is prone to arrhythmias, acute renal insufficiency, and shock.

AAAD lesions are usually extensive and often involve the branches and vessels of the celiac trunk and its surrounding organs. It is reported that the incidence of visceral poor perfusion before AAAD is 3.8%. [16] Our study found that patients with preoperative dissection involving the celiac trunk had a significantly higher risk of developing bad clinical outcomes. This is because when the dissection involves the celiac trunk and its branches, the false lumen formed will oppress the true lumen, resulting in occlusion of the branches supplying the liver. It is also possible that the branches of blood vessels are completely separated from the true cavity, resulting in different degrees of false lumen perfusion, which leads to hepatic perfusion disturbance and hypoxic-ischemic injury of liver tissue. The liver has enormous regenerative capacity. Thus, virtually complete restoration of normal liver structure and function can occur after hypoxic injury when normal hepatic perfusion is restored. [17] Repeated hypoxic injury, however, may lead to serious liver injury, further lead to many serious complications of others visceral system. Previous research found that patients with celiac trunk malperfusion before frozen elephant trunk for type A aortic dissection had a trend toward higher early postoperative morality rate. [18]

Long-term cardiopulmonary bypass will cause obvious damage to systemic organs, and great progress has been made in the study of its pathophysiological mechanism. Kumle et al. found that when the CPB time > 180 min, abnormal metabolic function of liver and the destruction of hepatocyte microstructure can occur. [19] Our results show that when the CPB time > 180 min, the risk of severe adverse outcomes in PHD patients increases significantly. Cardiopulmonary bypass is an abnormal physiological circulation in which non-pulsatile blood flow leads to reduced liver perfusion during operation. Some studies have reported that hepatic artery blood flow will be reduced by 20–45% during cardiopulmonary bypass, [20] and systemic blood flow will be redistributed, giving priority to the supply of lungs, brain and other important organs, further aggravating the low perfusion state of the liver, [21] resulting in hypoxic-ischemic injury of the liver. In addition, inflammatory mediators, microthrombi and cytotoxins produced during cardiopulmonary bypass aggravate SIRS, which will damage hepatocytes and further influence hemodynamics, coagulation system, immune system, vascular resistance and permeability as well as platelet concentration and function. [22]

Our study found that early postoperative albumin deceased was also one of the risk factors of poor prognosis in PHD patients after AAAD. Serum albumin, a stable protein synthesized in the liver and has been considered to be a powerful prognostic marker in the general population and many pathological settings, mainly as the result of malnutrition and inflammation. [23] Levey et al. had indicated that the serum albumin was also associated with in-hospital adverse outcomes and long-term mortality in patients with type B acute aortic dissection after endovascular therapy. [24] The mechanisms of poor prognosis association reduced albumin in PHD patients with AAAD are not clear. Previous research found low serum albumin most frequently arises from the inflammatory response, oxidative stress, and platelets aggregation. [25] The above pathological process is not only related to the physiological function of albumin, the effect of postoperative abnormal liver function on albumin synthesis, but also the pathogenesis and severity of aortic dissection. Serum albumin is also involved in maintaining plasma colloid osmotic pressure and reflecting the short-term nutritional status of the body. In our study, we found that patients with PHD after AAAD surgery with low early postoperative albumin were more likely to have adverse outcomes, suggesting that serum albumin levels are associated with poor prognosis.

In this study, we defined cutoff values of MELD-XI score (MELD-XI ≥ 14) for the diagnosis of PHD based on previous studies. A total of 153 cases (67.4%) met the diagnostic criteria for the early-stage biochemical markers after operation, which was similar to Wang and colleagues reported that an incidence of HD of 60.9% who received AAAD surgery. [26] Severe adverse outcomes occurred in 43.1% of postoperative HD patients and the hospital mortality rate was 12.4%. Previous research has shown that PHD was associated with increased early mortality and morbidity, but not with late death in midterm survival. [27] So, we tried to use MELD-XI score to stratify the high-risk patients with HD as an independent complication in the early postoperative period after AAAD surgery. This is further delineated by ROC analysis showing the optimal cut-off value of MELD-XI score for predicting severe adverse outcomes in patients with postoperative HD was 20.82 (sensitivity: 69.7%, specificity: 31.0%). Early detection of patients at high risk for early mortality and morbidity is the future focus. However, as a retrospective study with a single-center and a small-volume, the practical application value of the critical value is questionable. Therefore, prospective, multi-center studies are still needed to further enhance the persuasiveness.

To sum up, in patients with aortic dissection repaired by triple-branched stent graft, preoperative moderate/severe pericardial effusion, preoperative celiac trunk involvement, CPB time > 180 min and decreased early postoperative serum albumin were independent risk factors for severe adverse outcomes occurring in patients with PHD. For these AAAD patients with high risk factors, more attention should be paid to preoperative liver function evaluation and dynamic monitoring of postoperative liver and cardiovascular system function. Comprehensive strategies should be adopted during the operation, such as streamlining the operation, shortening the time of cardiopulmonary bypass, timely adjustment of the perfusion parameters of cardiopulmonary bypass, and strengthening the application of anti-inflammatory drugs. This will help ensure adequate blood perfusion of the liver during the operation and reduce hypoxic-ischemic injury to lower the risk of PHD.

The strength of the present study is that this was the first study aimed to identify the risk factors for severe adverse outcomes occurring in postoperative HD patients underwent AAAD surgery and adopt MELD-XI score to identifying high-risk patients which also excluded the influence of warfarin treatment.

## Limitation

The results should be interpreted by caution in other populations since all the data in this study was obtained from a single center, so a multicenter study is called to confirm these findings. Besides, there was a certain lag in the use of biochemical indicators to evaluate liver insufficiency, and there may be a certain degree of deviation.

## Conclusions

In conclusion, we confirmed that the PHD patients after AAAD surgery have higher early mortality and morbidity. Preoperative moderate/severe pericardial effusion, preoperative celiac trunk involvement, CPB time > 180 min and decreased early postoperative serum albumin were identified as independent risk factors for severe adverse outcomes in patients with postoperative HD. For these AAAD patients with high risk factors, attention should be paid to preoperative liver function assessment, intraoperative comprehensive management, and postoperative early intervention.

## Data Availability

The data that support the findings of this study are available on request from the corresponding author. The data are not publicly available due to privacy or ethical restrictions.

## Abbreviations

PHD: Postoperative hepatic dysfunction
CPB: Cardiopulmonary bypass
OR: Odds ratio
CI: Confidence interval
AAAD: Acute type A aortic dissection
MELD: Model for End-Stage Liver Disease
INR: International normalized ratio
LCOS: Low cardiac output syndrome
ECMO: Extracorporeal membrane oxygenation
TAR: Total arch repair
ACC: Cross-clamp
AUC: The area under curve
